# Supplementary figures and images for: Effect of a Multi-Strain Probiotic on Growth and Time to Reach Full Feeds in Preterm Neonates
Source: Nutrients. 2022 Nov 3;14(21):4658. doi: 10.3390/nu14214658 (PMC9659223; doi:10.3390/nu14214658)

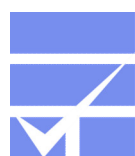

# CONSORT

TRANSPARENT REPORTING of TRIALS

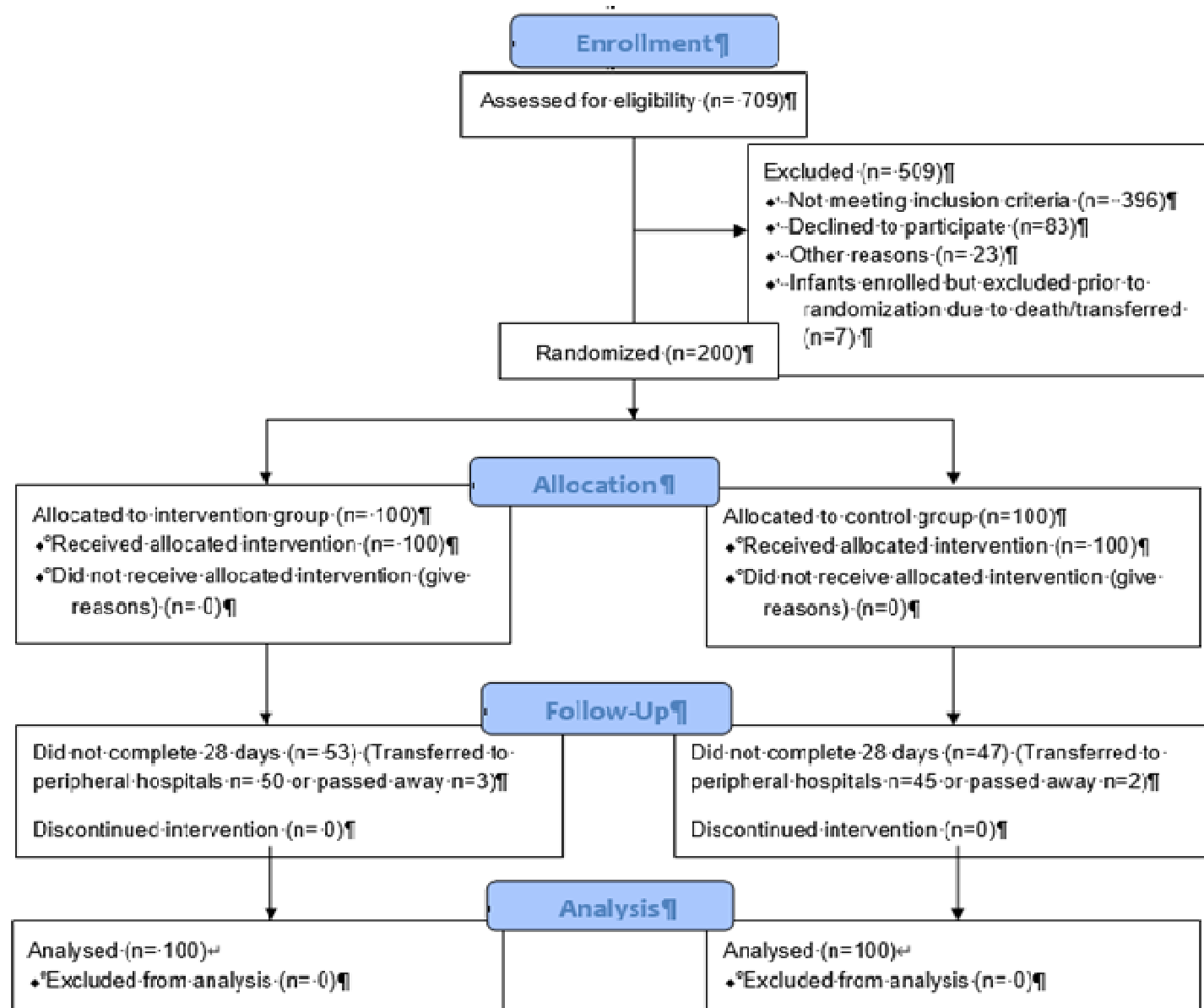

Figure S1. CONSORT 2010 Flow Diagram.

Supplement: Supplementary file 1 [file nutrients-14-04658-s001.zip › Figure S1.pdf]
